# Fluctuations in behavior and affect in college students measured using deep phenotyping

**DOI:** 10.1038/s41598-022-05331-7

**Published:** 2022-02-04

**Authors:** Constanza M. Vidal Bustamante, Garth Coombs, Habiballah Rahimi-Eichi, Patrick Mair, Jukka-Pekka Onnela, Justin T. Baker, Randy L. Buckner

**Affiliations:** 1 Department of Psychology, Harvard University, Northwest Science Building 280.05, 52 Oxford Street, Cambridge, MA 02138 USA; 2 Center for Brain Science, Harvard University, Cambridge, MA 02138 USA; 3 Department of Psychiatry, Harvard Medical School, Boston, MA 02114 USA; 4 Institute for Technology in Psychiatry, McLean Hospital, Belmont, MA 02478 USA; 5 Department of Biostatistics, Harvard T.H. Chan School of Public Health, Harvard University, Boston, MA 02115 USA; 6 Athinoula A. Martinos Center for Biomedical Imaging, Massachusetts General Hospital, Boston, Charlestown, MA 02129 USA

**Keywords:** Human behaviour, Risk factors

## Abstract

College students commonly experience psychological distress when faced with intensified academic demands and changes in the social environment. Examining the nature and dynamics of students’ affective and behavioral experiences can help us better characterize the correlates of psychological distress. Here, we leveraged wearables and smartphones to study 49 first-year college students continuously throughout the academic year. Affect and sleep, academic, and social behavior showed substantial changes from school semesters to school breaks and from weekdays to weekends. Three student clusters were identified with behavioral and affective dissociations and varying levels of distress throughout the year. While academics were a common stressor for all, the cluster with highest distress stood out by frequent report of social stress. Moreover, the frequency of reporting social, but not academic, stress predicted subsequent clinical symptoms. Two years later, during the COVID-19 pandemic, the first-year cluster with highest distress again stood out by frequent social stress and elevated clinical symptoms. Focus on sustained interpersonal stress, relative to academic stress, might be especially helpful to identify students at heightened risk for psychopathology.

## Introduction

First-year college students must navigate multifaceted change in their daily lives. In addition to completing demanding classes, assignments and exams, students must adapt to a different social environment, make new friends while also managing previous social relationships, and deal with roommates, finances, and health matters with increased independence from their childhood caregivers. These various academic, social, and personal demands, in addition to students’ oftentimes poor sleep and physical activity, can all contribute to psychological distress and increased vulnerability to mental illness^1–5^.

About one in three first-year college students present with at least one mental health disorder, most commonly depression and anxiety^6^, and an even larger proportion of students report experiencing psychological distress that affects their academic performance and day-to-day functioning^7,8^. Persistent psychological distress increases the likelihood of dropping out of college and also of engaging in self-injurious behavior, with potentially long-term consequences^1,9–11^. Especially as the share of individuals accessing higher education continues to increase^12^, understanding and addressing student mental health remains a pressing task.

Examining the nature and dynamics of students’ multidimensional affective and behavioral experiences can help us better characterize the correlates of psychological distress. The various demands associated with the transition to college life (academics, social relationships, and otherwise) are likely to have varying degrees of impact on students’ distress^5,13^. Moreover, the intensity of these demands is not uniform over time^14,15^, and their dynamics might help identify periods of heightened vulnerability to distress.

The increasing ubiquity of smartphones and wearables afford new opportunities to examine individuals at frequent sampling rates, for extended periods of time, and with relatively low participant burden^16,17^. Recent studies with students across college years have used these tools to collect a wide variety of passive sensing and survey-based measures over several weeks, including sleep, mobility, and studying and socializing behaviors. Researchers have adopted this deep phenotyping approach to describe behavioral patterns and predict students’ academic outcomes, stress levels, and depression symptoms^14,18–21^, and most recently to compare behavior and clinical symptoms before and after the start of the COVID-19 global pandemic^22^.

Here, we build on this emergent line of deep phenotyping research with two main goals. First, we sought to capture a full “year in the life” of a first-year college student by simultaneously examining multiple affective and behavioral experiences that have been previously associated with mental health outcomes. These include sleep, physical activity, academic and social behavior, and perceived stress levels and sources of stress (e.g., academics, social relationships, status, health, etc.). We were especially interested in assessing how these experiences fluctuate in relation to the academic calendar (e.g., during exams periods, extended school breaks, weekdays and weekends, etc.), and whether some experiences are more persistent than others across changing contextual demands. Our second goal was to explore individual differences in these experiences and their relation to mental health outcomes. Specifically, we sought to explore the presence of student subgroups with distinct affective and behavioral phenotypes, and how their distinguishing features relate to their respective levels of psychological distress.

To achieve these goals, we leveraged continuous wristband actigraphy data and daily smartphone-based self-report surveys in a sample of 49 first-year students for the full academic year, for a total of close to 10,000 daily observations. While emerging work combines passive data from several sensors to infer a wide range of complex behaviors and emotional experiences, here we limited our passive-sensing work to estimates with well-validated data processing and analysis pipelines, namely sleep detection and relative physical activity^23^. The rest of our metrics reflect participants’ daily self-reports of sleep quality and physical activity, stress levels and sources, positive and negative affect, and academic and social behavior. When the COVID-19 nationwide shut-down occurred two years later, we followed the same students again, seizing on the unique opportunity to prospectively assess their affective and behavioral patterns as they underwent the first three months of this unprecedented life transition.

Our results show substantial fluctuations in affect and behavior over the course of the year. For the average first-year student, these fluctuations followed the structure of the academic calendar, including higher stress at the beginning of the year and during exams periods, and marked changes from school semesters to school breaks and from weekdays to weekends. Clustering analyses revealed three student subgroups with multiple behavioral and affective dissociations and varying levels of distress throughout the year. A critical dissociation of the clusters was revealed by how frequently they endorsed stress sources related to social relationships (e.g., friends, family, etc.) as compared to sources related to academics (e.g., homework, grades). While academics were the most common source of stress for all, the cluster with highest distress stood out by their frequent social stress. These dissociations were observed again in the follow-up COVID-19 dataset. We identify frequent reports of social stress, in contrast to academic stress, as a helpful marker of current and subsequent psychological distress and clinical symptoms.

## Results

Table 1 presents summary statistics for all actigraphy- and survey-based metrics (for more details on missing data, see Supplementary Information and Supplementary Fig. 1). Below, we present the results of our two main research goals: (1) a description of the temporal dynamics of the average student’s daily stress, sleep, physical activity, and academic and social behavior, and (2) an exploration of student subgroups with distinct affective-behavioral phenotypes and patterns of psychological distress. We then extend these results with a 3-month follow-up study with the same students during the COVID-19 pandemic, allowing for the prospective replication of the student subgroup patterns.

**Table 1:** Sample descriptive statistics.

Metric	Range	M	Med	Between SD	Within SD
**Daily actigraphy: available observations per subject**	132–249	220	227	24.44	–
Sleep duration (hours)	0.28–16.05	7.28	7.26	0.54	1.54
Sleep timing regularity index	0–1	0.75	0.76	0.05	0.15
**Daily survey: available observations per subject**	98-255	202	216	47.32	–
Sleep quality	1–5	2.99	2.99	0.34	0.70
Energy	1–5	2.87	2.90	0.29	0.54
Physical activity	1–5	2.28	2.35	0.47	0.83
Time on schoolwork	1–5	2.42	2.38	0.42	1.06
Procrastination	1–5	2.27	2.09	0.68	0.84
Time on social interaction	1–5	3.34	3.36	0.53	0.91
Connected to others	1–5	3.14	3.13	0.47	0.66
Positive affect	1.00-5.00	2.65	2.63	0.49	0.59
Happy	1–5	2.90	2.86	0.56	0.76
Excited	1–5	2.55	2.47	0.59	0.81
Outgoing	1–5	2.56	2.54	0.49	0.79
Relaxed	1–5	2.58	2.49	0.51	0.85
Negative affect	1.00-4.86	1.82	1.78	0.47	0.46
Sad	1–5	1.84	1.79	0.57	0.77
Upset	1–5	1.76	1.58	0.52	0.76
Anxious	1–5	2.03	1.98	0.54	0.80
Lonely	1–5	1.78	1.65	0.68	0.68
Angry	1–5	1.38	1.22	0.39	0.52
Irritable	1–5	1.68	1.52	0.50	0.66
Self-dissatisfied	1–5	2.28	2.20	0.72	0.83
Stress	1–5	2.51	2.53	0.63	0.94
Number of daily stressors	0–15	2.75	2.32	1.80	1.65
Academic stress	0, 1	0.67	0.75	0.23	0.40
Social stress	0, 1	0.32	0.28	0.26	0.36
Status stress	0, 1	0.27	0.19	0.27	0.33
Global clinical severity score	25–76	44.68	44.43	9.25	5.21

*Range* range of scores observed in the study, *M* between-subjects mean, *Med* between-subjects median, *Between SD* between-subjects standard deviation, *Within SD* mean of within-subject standard deviation. Positive affect and Negative affect are composite scores of the indented items listed below them (see Methods).

### Sleep patterns show school break and weekly fluctuations

**Figure 1:**
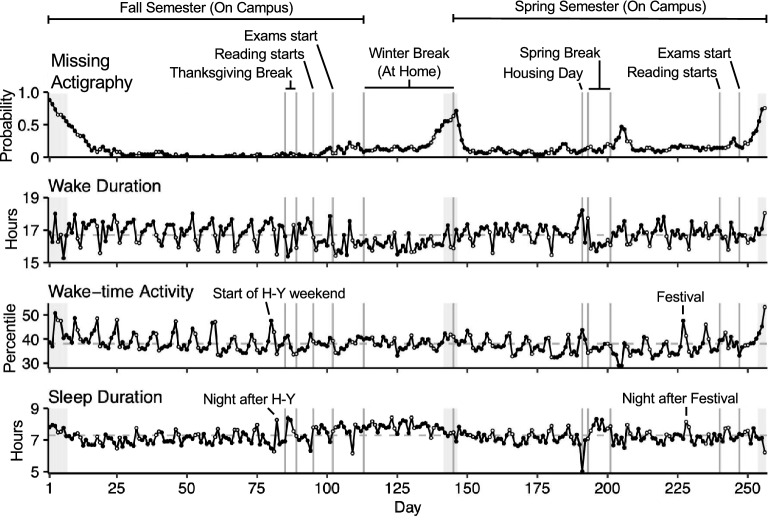
Activity and sleep patterns show school break and weekly fluctuations. Time series show daily actigraphy-derived observations averaged across the full sample. Vertical lines indicate landmark events in the academic calendar, labeled at the top. Black circles indicate Monday through Friday and white circles indicate Saturday and Sunday (sleep observations correspond to the day when the participant woke up). Dashed gray lines indicate the mean. The top panel shows the probability that survey-based observation is missing on any given day (i.e., the proportion of missing individual observations out of the total sample), and the gray shading across panels indicates days with more than 50% missing observations. *H-Y* Harvard-Yale football game, *Festival* music festival hosted on campus.

Broadly, there is a pattern of increased sleep and lower activity when students are released from structured academic demands. Group-averaged time series for daily actigraphy-derived sleep and wake-time activity metrics are shown in Fig. 1. The academic year has a Fall Semester and a Spring Semester (~ 16 weeks each) ending with Reading and Exams periods. During Reading period students work on assignments and prepare for final examinations. The two semesters are separated by a five-week class-free Winter Break. Additionally, there is a five-day Thanksgiving Break in Fall and a week-long Break in Spring.

Over Winter Break the average student had lower Wake-time Activity and longer Sleep Duration relative to the school semesters. Similar patterns were observed during Thanksgiving and Spring Breaks. Within the school semesters, Sleep Duration was longer during weekends relative to weekdays. Wake Duration and Wake-time Activity tended to peak on Fridays, the only day in the week when students are likely to both attend classes during the day and social events at night. In contrast, Wake Duration and Activity were the lowest on Sundays.

Social events also affected activity and sleep patterns. Wake-time Activity peaked at the start of the Harvard-Yale football game weekend, the day of the spring music Festival, and on Housing Day, when students received their housing assignments. Meanwhile, Sleep Duration showed peaks the night following the Harvard-Yale football game and the night following the spring music Festival, and it dropped sharply the night before Housing Day, when students are known to stay up late with friends prior to receiving their housing assignment. These dynamics reinforce that we captured real-world behavioral fluctuations relevant to daily student life.

### Behavior and affect also reveal marked fluctuations

**Figure 2:**
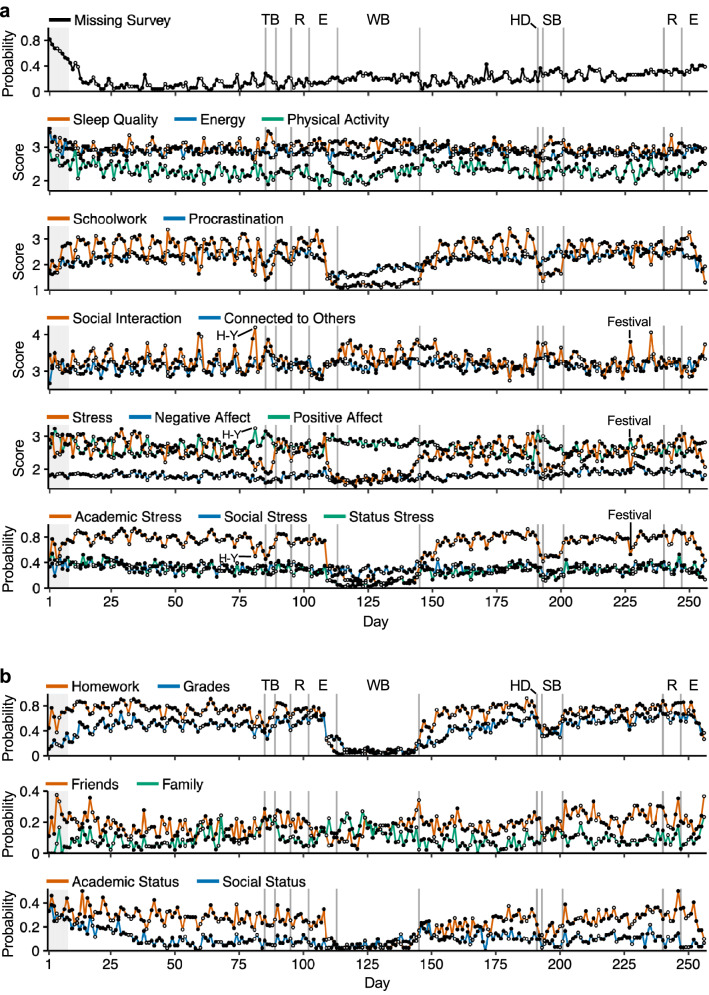
School-related fluctuations extended to various behavioral and affective domains. (**a**) Time series show survey-derived daily observations averaged across the full sample. Vertical lines indicate landmark events in the academic calendar, labeled at the top. Black circles indicate Monday through Friday and white circles indicate Saturday and Sunday. The top panel shows the probability that survey-based observation is missing on any given day (i.e., the proportion of missing individual observations out of the total sample), and the gray shading across panels indicates days with more than 50% missing observations. (**b**) Some of the individual stressor items that made up the composite academic, social, and status stress categories (displayed in the last panel of (**a**)) are shown in the first, second, and third panels, respectively. *TB* thanksgiving break, *R* reading period, *E* exams period, *WB* winter break, *HD* housing day, *SB* spring break, *H-Y* Harvard-Yale football game, *Festival* music festival hosted on campus.

Students experienced a period of markedly reduced Stress during Winter Break (Fig. 2a, Supplementary Fig. 2a). During this period they also reported slightly better Sleep Quality, spending less time on Schoolwork, more time Interacting with others, and fewer daily stressors. Decreases in Stress and increases in Positive Affect and Social Interaction extended to Thanksgiving and Spring Breaks, to days with university-wide social events, and to weekend days within the school semesters (Fig. 2a, Supplementary Supplementary Fig. 2b). Thus, at the group level, structured academic demands and releases from them are associated with large fluctuations in behavior and experienced distress.

Of note, not all items showed clear school-related changes: Energy, feeling Connected to others, Negative Affect, and Social Stress showed similar levels from school semesters to Winter Break, and from weekdays to weekends. As will be illustrated later, there were individual differences tied to clinical symptoms of distress that are distinct from those tied closely to academic demands.

### Academics is the most common stressor but social stress is relentless

Stress was commonplace and had multiple sources (Fig. 2b). Academic stress sources were the most frequently reported during the school semesters. Homework-related stress, in particular, was two to eight times more frequent than stressors related to social relationships or status. However, Academic Stress was situational, with a near complete attenuation during school breaks.

Social Stress was less common but displayed a distinct sustained pattern. The probability of experiencing Social Stress from Friends and Family was present throughout the year, including during school breaks. Friends were the most frequent source of Social Stress during the school semesters. Family stress increased during the school breaks (when students usually go back home), reaching a similar and at times higher probability than Friend-related stress.

### Latent states of distress reflect the structure of the academic calendar in the group

The shared fluctuations in behavior and affect described above were summarized as latent distress states using a multivariate hidden Markov model (HMM) over the group-averaged time series. The three-state model had the lowest Bayesian Information Criterion (BIC) (Supplementary Table 1). The three states identified by the model were intuitive and reflected a progression from lowest to highest distress with overall increasing mean values for Stress, Negative Affect and Energy, and decreasing mean values for Sleep Quality and feeling Connected to others. We refer to these states as Lowest, Medium, and Highest Distress states (these labels are relative; e.g., the “Highest” distress state had the highest mean value for feeling Stress, but this value corresponds to “moderate” stress on the survey scale).

**Figure 3:**
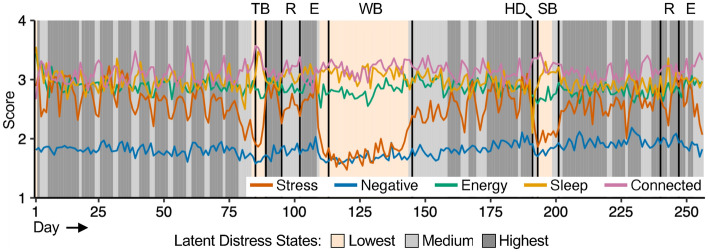
Latent states of distress reflect the structure of the academic calendar. Latent states were estimated via multivariate hidden Markov modeling over the group mean time series of stress, Negative affect, Energy, Sleep quality, and feeling Connected to others. *TB* thanksgiving break, *R* reading period, *E* exams period, *WB* winter break, *HD* housing day, *SB* spring break.

The estimated sequence of Latent Distress States over the year revealed a clear, structured pattern of distress that reflects academic demands (Fig. 3). The semesters were characterized by the Medium and the Highest Distress levels, with a weekly pattern: Medium Distress on Fridays and Saturdays, and Highest Distress the remainder of the week. The Lowest Distress state was present during the extended breaks including Thanksgiving, Winter, and Spring Breaks.

### Clustering analysis identifies three distinct student profiles

**Figure 4:**
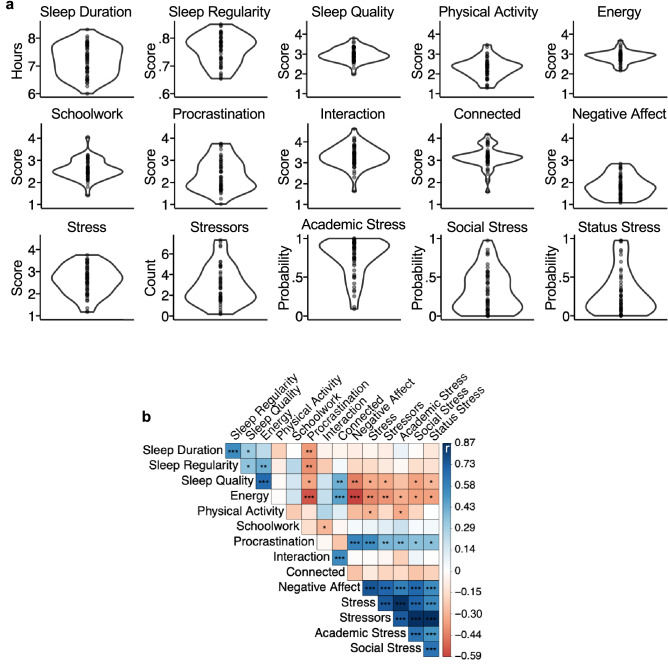
Between-person variability was substantial and showed latent structure. (**a**) Violin plots show distribution of participant-averaged metrics. (**b**) Correlation matrix shows between-person Pearson *r* correlation coefficients; stars represent statistical significance (*p < 0.05, **p < 0.01, ***p < 0.001).

Not all students experience their first year the same way. Descriptive analysis revealed substantial between-person variability in most affective and behavioral measures (Fig. 4a). Moreover, moderate to large correlations between measures illustrated that there might be significant latent structure (Fig. 4b). For example, Negative Affect, Stress, Academic Stress and Social Stress all showed strong positive correlations.

**Figure 5:**
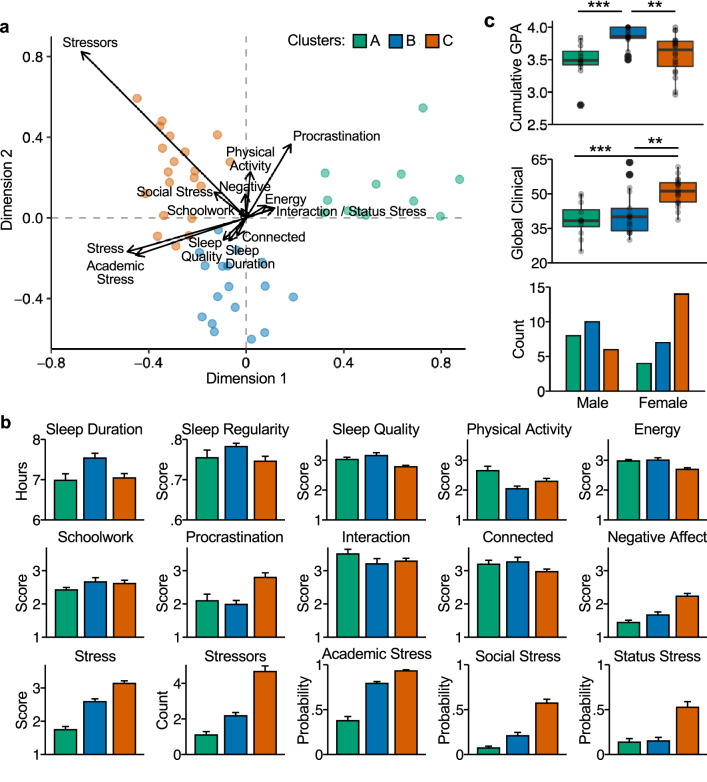
Clustering analysis reveals three distinct student profiles. (**a**) Biplot shows both the maximal separation among the clusters, displayed as dots colored by cluster, and the original variables used in the analysis, shown as standardized basis vectors on the two dimensions. The length of the vectors represents standardized regression coefficients. The arrows for Interaction and Status Stress are almost fully overlapping, and the vector for Sleep Regularity is almost exactly at (0, 0). (**b**) Bar plots show between-person means of each clustering variable separately by cluster. Error bars represent standard errors of the mean. (**c**) Boxplots show participants’ cumulative grade point average (GPA) their first year of university (top) and mean Global Clinical symptom severity (middle). Asterisks represent statistically significant differences between cluster pairs (two-tailed *t*-tests; **p < 0.01, ***p < 0.001). Bottom bar plot shows the self-reported sex breakdown of the three clusters.

Clustering analysis was employed to explore individual differences. A latent profile clustering analysis indicated that the three-cluster mixture was the best solution. The clustering structure is visualized in Fig. 5a (for more details on this biplot, see Supplementary Information). The first dimension (62% of the eigenvalues) separated Cluster A (comprising 12 participants) from the other two clusters, along measures including Stress, Academic Stress, and number of daily Stressors, with Cluster A showing the lowest values on these variables. The second dimension (38% of the eigenvalues) separated Cluster B (17 participants) from Cluster C (20 participants), with the former showing lower values on measures including Procrastination and higher values for Sleep Duration and Sleep Quality. This second dimension also separated Cluster B from the other two clusters along Physical Activity, with Cluster B showing the lowest values.

The between-subject mean values of each clustering variable by subgroup are illustrated in Fig. 5b. On average, participants in Cluster A reported the lowest scores for Negative Affect, Stress, Social Stress, Academic Stress, and time spent on Schoolwork, and the highest levels of Physical Activity. On the other end of the spectrum, participants in Cluster C reported feeling the most Negative Affect and Stress, the highest number of daily Stressors, medium to high probability to experience Academic, Social, and Status Stress, the lowest Sleep Quality, lowest Energy, and highest Procrastination.

Cluster B was intermediate in terms of Stress but additionally stood out on other measures. Participants in this cluster had the highest Sleep Duration and the lowest Physical Activity. Like Cluster A, they reported high levels of time spent on social Interaction and feeling Connected to others, and low Negative Affect. But in contrast to Cluster A, Cluster B reported elevated Academic Stress. Crucially, this stress was selective to Academics, with low Social and Status Stress. In this sense, Cluster B is much like Cluster A in terms of general distress but has elevated distress focused around academic demands. By contrast, Cluster C has a pattern of distress that is pervasive across academic and social domains.

### Student profiles associate with academic performance and clinical symptoms

To test whether separation of the participants into three subgroups was predictive of additional data, we turned to independently-acquired assessments of academic performance (first-year cumulative grade point average, GPA) and psychological distress as reflected by the Global Clinical Score (Fig. 5c). Cluster B, which had a high probability to report Academic stressors (but not other stress sources), had the highest cumulative grades (GPA; ANOVA *p* < 0.001; post hoc* t-*tests of Cluster B vs. Cluster A and Cluster B vs. Cluster C, ps < 0.01). Most critically, Cluster C, which had the highest scores for Negative Affect, Stress, and both Academic and Social Stress, also had the highest Global Clinical Scores (ANOVA *p* < 0.001; post hoc* t-*tests of Cluster C vs. Cluster A and Cluster C vs. Cluster B, *p*s < 0.01). Women were more likely to be in Cluster C relative to the other two groups, while men showed an even distribution across the clusters. These sex differences are in line with the well-documented higher rates of anxiety and mood disorders among females compared to males^24–26^.

### The three student profiles show distinct distress dynamics

**Figure 6:**
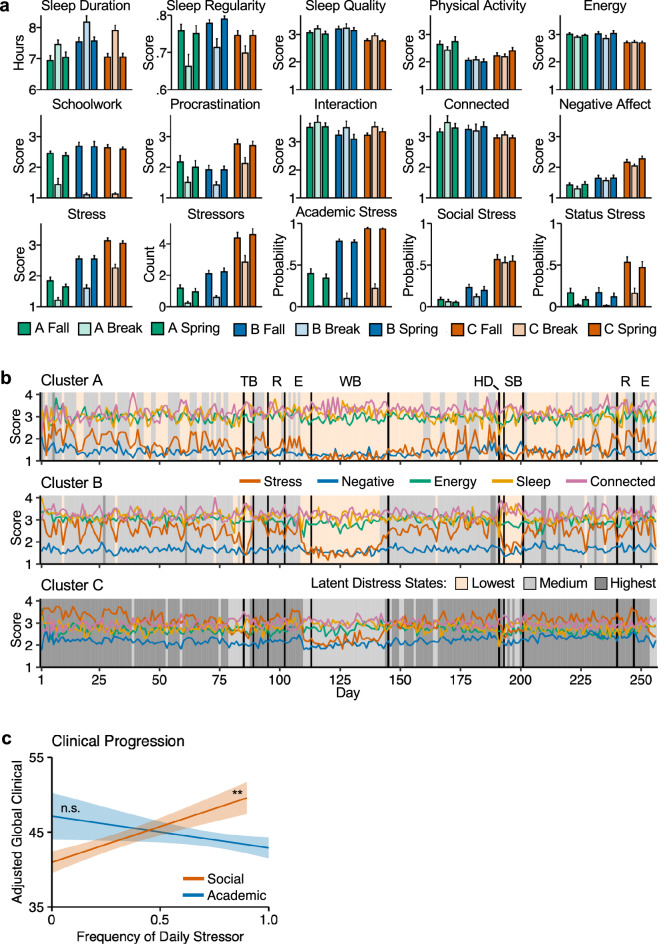
The three student profiles show distinct distress dynamics. (**a**) Bar plots show between-person means of each variable separately by student profile and by term. Error bars represent standard errors of the mean. (**b**) Sequence of most likely latent states of Distress for the three student profiles. *TB* thanksgiving break, *R* reading period, *E* exams period, *WB* winter break, *HD* housing day, *SB* spring break. (**c**) Frequency of Social stressors, but not Academic stressors, prospectively predicts subsequent global clinical symptoms. **Statistical significance at p < 0.01, *n.s.* not statistically significant (p > 0.05).

To further understand how the year was experienced by the distinct subgroups of participants, the measures were graphed separately for each of the major academic year epochs: Fall Semester, Winter Break, and Spring Semester. All three of the subgroups showed clear differences between the School semester versus the Break (Fig. 6a) (as well as weekday versus weekend patterns; Supplementary Fig. 3). However, while even Cluster C showed decreased Negative Affect and Stress scores during Winter Break and weekends, between-cluster differences were robust and present throughout the year. Notably, Cluster C’s Procrastination, Negative Affect, and Social Stress scores during Winter Break were higher than Clusters A and B’s levels of the same variables during the School terms.

We used a three-state multilevel HMM to examine if the sequence of latent Distress states identified at the group level (shown in Fig. 3) differed by cluster (Supplementary Table 2).

What emerged were distinct patterns of distress across the three subgroups (Fig. 6b). Cluster A was in the Lowest or Medium Distress states almost all the time including during the academic semesters and exam periods. Cluster A’s marginal probability to be in the Highest Distress state remained near zero throughout the year (Supplementary Fig. 4). Cluster B was in the Medium Distress state for most of the academic semester and reached the Highest Distress state on a handful of days around periods of midterm examinations, consistent with their distress being associated with academic demands. Finally, Cluster C was in the Highest Distress state during most of the school semesters, dropping to the Medium distress state during the school breaks. This group did not reach the Lowest Distress state at all, with its marginal probability to be in the Lowest Distress state remaining at zero throughout the year (Supplementary Fig. 4).

### Social stress, but not academic stress, predicts clinical symptoms

An interesting feature of the above descriptive analyses is the contrast between Academic and Social Stress sources in relation to their temporal dynamics and correlation to clinical symptoms. While Academic Stress is the most robust stressor experienced by the participants, the cluster with the highest Global Clinical Score stands out by their higher frequency of Social Stress. Social Stress was less common than Academic Stress but was also less situational. For example, as seen in Fig. 6a, while Cluster C’s probability to report Academic Stress reduces during Winter Break, their probability to report Social Stress remained stable throughout the break. These observations suggested that frequent report of interpersonal stressors might be a more sensitive marker of clinical symptoms than academic stressors.

A post hoc mixed effects linear model including Social Stress and Academic Stress as predictors of Global Clinical Scores sought to formally test this observation across all participants. The frequency of Social Stress during the semester was a statistically significant predictor of subsequent Global Clinical Scores at the end of the semester (*B* = 9.56 (3.27), *t* = 2.92, *p* < 0.01), above and beyond baseline symptoms and Academic Stress (Fig. 6c). In contrast, Academic Stress was not a statistically significant predictor of Global Clinical Scores (*B* =  − 4.24 (3.98), *t* =  − 1.06, *p* = 0.29) when controlling for Social Stress. As revealed in the next section, this pattern of dissociation continued in independent, prospectively acquired data when the same participants were followed years later during the COVID-19 pandemic.

### Student profiles prospectively predict experiences during the COVID-19 pandemic

**Figure 7:**
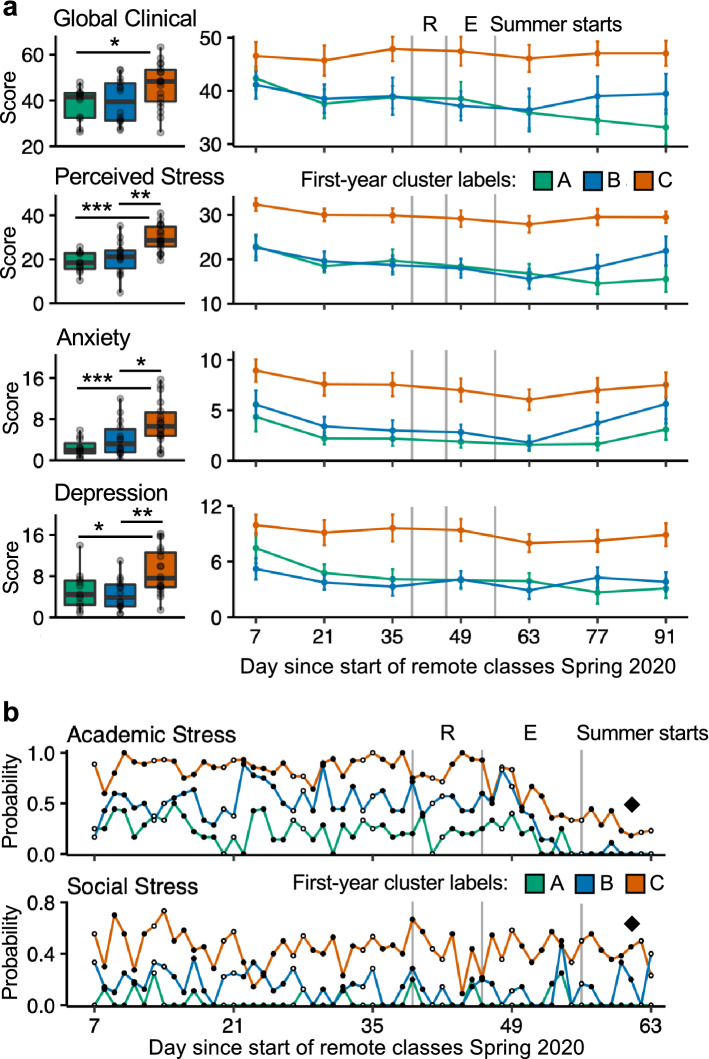
Student profiles prospectively predict experiences during the COVID-19 pandemic. (**a**) Boxplots on the left represent participant-means throughout the follow-up study period, grouped by the cluster labels assigned in the first-year study. Asterisks represent statistically significant differences between cluster pairs (two-tailed *t*-tests; *p < 0.05, **p < 0.01, ***p < 0.001). Time series on the right show cluster averages per each biweekly questionnaire timepoint. Error bars represent standard error of the mean. (**b**) Time series show daily observations averaged by first-year cluster labels. Diamonds emphasize the different trajectories of Academic versus Social Stress for Cluster C into Summer Break. *R* reading period, *E* exams period.

The outbreak of the COVID-19 pandemic posed a unique opportunity to assess the stability of our observations under a new stressful life transition. In March 2020, now in their third year of college, 43 out of the original 49 students (88% retention), enrolled in a fully remote three-month follow-up study. Surprisingly, there was no statistically significant difference in participants’ Global Clinical Scores between the first-year study and the follow-up study (paired *t*-test, *p* = 0.07). Analyses focused on the student subgroups determined in the first-year data. What emerged is that the three subgroup clusters continued to have distinct patterns of distress, with Social Stress, but not Academic Stress, indicative of those individuals with severe distress (Fig. 7).

Cluster C, which had originally shown the highest overall distress, again stood out with elevated Global Clinical Scores during the COVID-19 pandemic (Fig. 7a; ANOVA *p* < 0.05; post hoc* t-*test of Cluster C vs. Cluster A, *p* < 0.05; post hoc* t-*test of Cluster C vs. Cluster B, *p* = 0.06) and higher Perceived Stress and Anxiety and Depression symptoms (ANOVAs ps < 0.01; post hoc* t-*tests of Cluster C vs. Cluster A and Cluster C vs. Cluster B, *p*s < 0.05). By contrast, Clusters A and B both reported low Perceived Stress and clinical symptoms (post hoc* t-*tests of Cluster A vs. Cluster B, *p*s > 0.11). Moreover, while at the beginning of the pandemic all three clusters showed similar levels of Global Clinical Scores, Clusters A and B’s overall trajectories showed decreases over time, but Cluster C’s remained consistent throughout.

Examination of the dynamic patterns for Academic and Social Stress again presented dissociations in this follow-up period (Fig. 7b). Just like in their first year of college, the probabilities to report Academic Stress and Social Stress were both high for Cluster C and both low for Cluster A, while Cluster B’s probability to report Academic Stress was high relative to their low probability to report Social Stress. Furthermore, a critical observation comes from the trajectories of Academic and Social Stress: while the probability to report Academic Stress decreases substantially for all clusters toward the end of the final Exams period, Cluster C’s report of Social Stress remained elevated even into the beginning of summer break. The relentlessness of Cluster C’s Social Stress mirrors the stability of this cluster’s clinical scores, thus further pointing to Social Stress as the critical marker.

## Discussion

The current study used a year-long deep-phenotyping approach to measure actigraphy-derived sleep behavior and daily self-reported metrics of affect and sleep, academic, and social behavior in a group of 49 first-year college students. Our results provide new insights into the variability of students’ affective and behavioral experiences, both over time and across individuals, and their relationship to psychological distress.

Students’ behavior and affect fluctuated with academic demands, in line with results from previous deep-phenotyping studies with college students^15,18,22^. Stress levels were highest during the first few weeks of the academic year as well as during midterm and final exams periods. On school breaks, as well as on Fridays and Saturdays, the average student slept more, spent less time on schoolwork and more time interacting with others, felt less stress, and reported fewer academic- and status-related stressors compared to the school semesters and weekdays.

Not all participants experienced their first year of college the same way. Our clustering analysis identified three student subgroups with different affective and behavioral features and varying levels of global psychological distress on an external clinical inventory. A critical dissociation of the clusters was revealed by how frequently they endorsed social sources of stress (friends, family, roommate, and/or partner) compared to academic sources of stress (schoolwork and/or grades). These patterns replicated in the follow-up COVID-19 study. While academic stress was more frequently reported than social stress for all groups, the cluster with elevated clinical scores stood out by frequent social stress. The other two clusters had varying degrees of academic stress, but both had infrequent social stress and low clinical scores. In a post hoc mixed effects model further probing this dissociation, the frequency of social stress, but not academic stress, was a significant predictor of subsequent clinical scores at the end of the semester. Although our analyses did not systematically test all possible predictors of psychological distress, these observations suggest that, when it comes to sources of stress, academics are a robust, normative stressor for most students, while a focus on persistent interpersonal stress might be especially helpful to identify those at risk for psychopathology.

These results are in line with previous literature showing academics as a frequently reported source of stress among students^2,3,27^, and pointing to interpersonal stressors as more strongly associated than non-interpersonal ones with mental health outcomes (e.g., depression symptoms^27–29^). Social relationships are at the core of individuals’ development and daily experience and play an important role in shaping psychological and emotional wellbeing. Social relationships can represent a critical source of support, but they can also represent a source of stress, either due to direct conflict (e.g., having an argument with friends or family) or to more indirect or subjective pressures and aggravations (e.g., worrying about others’ expectations, lack of personal space due to roommates, etc.). While occasional social conflict and hassles are inevitable and important for the development of social skills, our results suggest that sustained interpersonal stress, at least as perceived and measured through subjective report, is associated with greater psychological distress and clinical symptoms.

Our findings do not imply that academic stress is irrelevant to students’ psychological wellbeing and mental health. For example, the cognitive demands of academics might exacerbate interpersonal stress by making students more sensitive to social friction. However, frequent academic stress, by itself, does not associate with heightened clinical symptoms of distress. Cluster B had frequent reports of academic stress, but low daily negative affect and low psychological distress, as well as the highest grades among the three clusters. One possibility is that these students perceived academic stressors as a motivating challenge under their control rather than as an overwhelming pressure. Cluster C had frequent academic stress as well as the highest negative affect and psychological distress scores, and it is possible that academic stress contributed to their distress, but it was their level of social stress that set them apart.

While our group-level results suggest that an early focus on identifying, resolving, and preventing interpersonal stress might be especially important to help reduce students’ risk for psychological distress, assessing each participant’s data separately might provide more tailored recommendations. For example, future analyses could leverage our intensive longitudinal design to provide individualized models of psychological distress, using lead-lag analyses to assess the directionality of associations among stress and negative affect over days or weeks, and assess whether certain behaviors (e.g., sleep or physical activity) influence the relationship between a daily stressor (social, academic, or otherwise) and negative affect.

The meaningful structure evidenced extensively in the data validate the use of digital phenotyping tools to capture a wide range of behavioral metrics relevant to student life over extended periods of time. However, some limitations should be noted. Most enrolled participants were highly compliant, but there were non-trivial amounts of missing data, especially among the daily phone-based surveys toward the last few months of the study. Although missingness in the daily assessments were not associated with participants’ global clinical scores, it is difficult to know how different the results would look with perfect completion rates. Payment structures and phone notifications were designed for participant compliance and retention, but future work should explore additional strategies.

Our analyses relied heavily on self-report. While we were interested in participants’ subjective perception of stress and affect, participants’ report of their behavior (e.g., how much time they spent on schoolwork or interacting with others) might be inaccurate. Participants’ answers to these questions showed meaningful patterns (e.g., spending less time on schoolwork and more time interacting during weekends and school breaks), but objective metrics derived from validated passive sensing pipelines might provide greater accuracy^18,19^.

Finally, our sample was a small group of students living on the campus of an elite United States university, and might not be representative of all first-year college students. For example, if applied to larger or more diverse samples, our analyses would likely identify different and/or more student phenotypes. Our finding that frequent interpersonal stress is more closely associated with psychological distress than academic stress also warrants further examination in larger samples.

### Conclusion

The transition to college involves a wide range of academic, social, and physical demands, all of which can contribute to students’ psychological distress and increased vulnerability to mental illness. Our year-long deep-phenotyping investigation provides new insights into the correlates of psychological distress by examining the nature and dynamics of these multidimensional affective and behavioral experiences. Our results revealed substantial variability, both over time and between individuals, in students’ stress levels and sources, sleep, academic and social behavior. Academic stress was common for most students. Those with highest psychological distress stood out by their frequent report of stressors related to social relationships. Although further research is needed in larger and more diverse samples, interpersonal stressors might represent a useful marker of distress and potential target for interventions seeking to support students’ college adjustment and overall wellbeing.

## Methods

### Participants

First-year students living on campus at an elite university in the United States were recruited within the first two weeks of their Fall semester for a year-long study via flyers and e-mail. Participants were required to be enrolled full-time in classes and own an Apple iPhone or Android smartphone compatible with the smartphone application used to collect daily surveys, Beiwe, which is part of the open-source Beiwe platform for digital phenotyping^30^. Participants were not excluded for psychiatric disorders or medication use. Interested participants scheduled an in-person consent session where study procedures were explained. Informed consent was obtained from all study participants. All study methods were approved by the Institutional Review Board of Harvard University and were performed in accordance with the relevant guidelines and regulations.

From an initial recruitment pool of 68 individuals, 19 were excluded from analysis based on issues with data acquisition including early withdrawal from the study (n = 7), technical failure of the actigraphy data (n = 1), poor quality actigraphy data (n = 2), and completion of < 100 daily surveys across the data collection period (n = 9). The final cohort was N = 49 (ages 18–19, mean age = 18.06; 25 female). Of this final sample 63% identified as White, 14% Black, 10% Asian, 4% American Indian, and 4% Mixed-Race. Twelve percent of the sample reported prior diagnosis of a psychiatric disorder (including anxiety, depression, and/or ADHD), of which 66% were active diagnoses. First-year college students have not yet declared their area of study, but participant-reported intended future occupation was 31% medicine, 14% business or finance, 12% academia or other research, 10% engineering, 10% policy or government, 8% law, and 6% other or undecided. Finally, 94% of participants were iPhone users and 6% were Android users.

### Study design

This intensive longitudinal observational study collected data over the full academic year and a few days into the summer break. Participation involved completing a battery of online questionnaires at the beginning, middle, and end of the study, completing smartphone-based daily surveys and a voice-recorded diary, wearing an actigraphy wristband for continuous activity and sleep monitoring for the duration of the study (GENEActiv Original, Activinsights Ltd., Kimbolton, UK), and brief in-person check-ins every 3–4 weeks. Participants were compensated per hour for online surveys, $1 per each daily survey they submitted, and $1 per day for continuously wearing the actigraphy wristband. They were also given a milestone bonus for completing half of the study and a larger milestone bonus for completing the full study.

### Measures and quality control

#### Objective activity and sleep measures

Sleep duration, sleep timing regularity, and wake-time activity were derived from the accelerometer data. Participants were instructed to wear the wristband on their nondominant wrist continuously, including during sleep and when bathing. The wristband collected tri-axial acceleration with precise timestamps at a rate of 30 Hz while participants were on campus, and 10 Hz while participants were away for winter break (in order to extend battery life and memory while participants were not on campus). Participants were instructed to press the wristband’s button when they began trying to sleep at night and immediately after they awoke in the morning. Following the initial consent and receipt of the wristband, individuals exchanged their wristband for a fully charged one with reset memory at the in-person check-ins.

The DPSleep processing pipeline^23^ was applied to the raw actigraphy data to automatically estimate minute-based activity and to detect the major sleep episode for each day. The pipeline first removes detected minutes when the individual was not wearing the device using the tri-axial acceleration variance, and then proceeds with its estimation. Days where one of the boundaries of the sleep episode (i.e., rises in relative activity both before and after a period of lower activity) could not be detected due to missing data were labeled as unusable. Two trained independent raters examined the automatically detected start and end times and usability label of each sleep episode against the minute-based activity levels and the participant button presses. If necessary, they adjusted the automatic times and labels. A full description of the processing pipelines applied to the actigraphy data, including quality control steps, can be found in Ref.^23^.

All data that passed quality control was included in analysis, including days with no detected sleep episode (i.e., with no extended periods of lower relative activity). The following metrics were derived and used in analyses:

##### Sleep duration

Daily sleep duration reflects the number of hours between the timestamps for the start and end of the day’s longest detected sleep episode.

##### Sleep timing regularity

Daily sleep regularity reflects the proportion (from 0 to 1) of overlap in sleep timing between each study day and the participant’s average sleep timing. See Ref.^23^ for details. An individual who sleeps and wakes at the same time on both study day *j* and on their average sleep day would get a score of 1 for study day *j*. Conversely, if the sleep episode is completely non-overlapping with their average sleep day, the daily score would be 0.

##### Wake-time activity

Daily wake-time activity is the average of the minute-based, person-normalized activity percentiles (estimated by DPSleep) across the wake period (i.e., the period between two detected sleep episodes in consecutive days). If daily sleep episode data was missing, wake-time physical activity was also marked missing.

#### Daily phone-based surveys

Smartphone surveys were administered via the Beiwe application^30^. Each night, before they went to sleep, participants completed a 46-item self-report survey related to their daily lives. Questions were designed to assess a broad range of behaviors and internal states over the past 24 h, including general physical health, daily consumption habits, positive and negative affect, studying behaviors, stress levels and sources, and sociability and support^31^. Most questions were answered using a 5-point Likert scale. We limited our analyses to a subset of questions selected a priori that probed the range of affective and behavioral experiences stated in our research goals. Specifically, these questions probed subjective sleep quality (from “Terrible” to “Exceptional”), energy levels and physical activity levels (from “Very little or not at all” to “Extremely”), how much of their awake time they spent on schoolwork and socializing (from “0–20% of my time” to “80–100% of my time”), how much they felt stress (from “Very little or not at all” to “Extremely”), positive affect (individual items for happy, outgoing, excited, and relaxed) and negative affect (individual items for sad, upset, hostile, irritable, lonely, anxious), and participants’ sources of stress (selected from a checklist spanning academics, social relationships, status, health, and financial situation categories). A full list of the daily survey questions used in the present analysis and other survey details are included in the Supplementary Information.

Surveys submitted between 5PM (local time) the day the survey opened and 6AM the following day were considered on time. Surveys submitted past 6AM the day after the survey went live were discarded and marked as missing. A participant was included in analysis if they were compliant with at least 100 daily surveys across the entire data collection period, and only on-time surveys from those participants were included.

#### In-person and periodic web-based surveys

All participants completed basic demographic and physical and mental health questionnaires at their baseline in-person visit. The health questionnaire asked participants to report whether they had past or current diagnoses of a series of conditions listed on a checklist (Supplementary Information). Additionally, participants used REDCap, a secure online platform^32^ to complete a clinical questionnaire (Symptoms Checklist 90 Revised, see below)^33,34^, other surveys (not analyzed here), and report their cumulative grade point averages (GPA). This REDCap-based survey battery was collected at three timepoints in the year: within the first month of enrollment (baseline), at the midpoint of the study period (during Winter Break after Fall semester final grades had been returned) and at the end of the study period (after Spring semester grades had been returned).

##### Symptoms Checklist 90 Revised

The SCL-90-R^33,34^ is a 90-item self-report questionnaire that assesses the severity of a broad range of psychological problems and clinical symptoms, including somatization, internalizing, psychoticism, and other domains. Each question asked participants to indicate how much they were bothered by that problem during the past two weeks using a 5-point Likert scale, from “Not at all” (0) to “Extremely” (4). The Global Severity Index (GSI) is a subscale of the SCL-90-R that reflects the overall current level or depth of distress in terms of both number of symptoms endorsed and intensity of distress^33^. The GSI, which we also refer to as Global Clinical Score in this paper, was computed for each participant at each of the three timepoints, transformed to adolescent- and gender-normed T-scores, and then averaged to obtain a single, person-level summary score of global psychological distress (possible range following T-score transformation = 25–81). Symptoms are considered to be at clinical levels if the GSI T-score is greater than 63^33^. The GSI subscale has good sensitivity and reliability in psychiatric and non-psychiatric populations^35,36^, and had good internal consistency in all three timepoints in the first-year study (Cronbach’s α = 0.87).

#### COVID-19 prospective data collection during the COVID-19 pandemic

In March 2020, the COVID-19 global pandemic prompted the closure of the university’s on-campus activities. The following week, the 49 original participants were recontacted (now in their third college year) and invited to participate in a fully remote, 13-week follow-up study. Participation involved completing computer-based survey batteries via REDCap every two weeks and smartphone-based daily surveys via Beiwe. Wristband-based actigraphy data was not collected. Forty-three of the 49 year-long study participants enrolled in the new study (i.e., 88% retention rate, with > 82% retention within each of the three student clusters. Three of the 43 participants provided only the biweekly surveys). The other six original participants (one from Cluster A, three from Cluster B, and two from Cluster C) declined to participate or did not respond to our contacts. Data were collected starting the week after Spring Break for a total of 94 days, capturing the second half of the Spring semester (seven weeks) and six weeks into the summer break. Thirty-two percent of the sample (up from 12% in first-year year study) reported a lifetime psychiatric disorder diagnosis. Participants were compensated $10 for each completed biweekly survey and $1 for each daily survey, as well as given a milestone bonus for completing the study, which was scaled to reward few missed surveys. All participants were re-consented. All study procedures were approved by the Institutional Review Board of Harvard University and were performed in accordance with the relevant guidelines and regulations.

Biweekly survey batteries included the SCL-90-R (same as described in the year-long study; GSI subscale had good internal consistency in all timepoints in the follow-up study, Cronbach’s α = 0.85–0.91) as well as the following assessments:

##### Perceived stress

Perceived stress was measured with the Perceived Stress 14-item Scale (PSS-14^37^). Participants indicated how often they felt the way described in the items using a 5-point scale (from 1 = “Never” to 5 = “Very often”), with higher scores indicating higher perceived stress. Answers to all items are summed to provide a total perceived stress score. The PSS-14 has been found to have good validity and reliability^37,38^, and had good internal consistency in all timepoints in the follow-up study (Cronbach’s α = 0.85–0.91).

##### Anxiety

Anxiety symptoms were measured with the Generalized Anxiety Disorder 7-item scale (GAD-7), which queries symptoms occurring in the last two weeks. Items are scored on a Likert scale ranging from 0 to 3 and summed to create a symptom severity score. The GAD-7 has good reliability and validity^39^, and had good internal consistency in all timepoints in the follow-up study (Cronbach’s α = 0.88–0.91).

##### Depression

Depression symptoms were measured with the Patient Health Questionnaire 9-item scale (PHQ-9), which assesses depression symptoms occurring in the last two weeks. The item assessing suicidal ideation was not included in the surveys. Items are scored on a Likert scale ranging from 0 to 3 and summed to create a symptom severity score. The PHQ-9 has good reliability and validity^40^, and had good internal consistency in all timepoints in the follow-up study (Cronbach’s α = 0.83–0.87).

### Analytical approach

#### Daily survey-derived composite scores

Daily-level composite scores were derived for Positive Affect, Negative Affect, and the stress source categories using items from the daily phone-based surveys. Individual items for Happy, Excited, Relaxed, and Outgoing were averaged into a composite Positive Affect score, and individual items Sad, Upset, Anxious, Irritable, Angry, Lonely, and Self-Dissatisfied were averaged into a composite Negative Affect score. Individual items within each composite score had positive same-day correlations (Supplementary Fig. 5). Additionally, we computed binary scores at the daily level to indicate whether the participant reported any of the stressors under three categories: Academic Stress (individual items for Homework, Grades), Social Stress (individual items for Friends, Family, Roommate, Partner), and Status Stress (individual items for Academic Standing, Social Status). Finally, we computed a daily Stressors variable that represents the sum of all individual stressors endorsed by the participant.

#### Group-averaged year-long time series and structure of the academic year

To compute group-level time series of each of the metrics of interest, data were averaged at the daily level (from the first day of the Fall semester to the last day of the Spring semester, i.e., 256 days) across all available participant observations in the final sample. Days with more than 50% missing observations across the sample are indicated with gray shading in the relevant figures.

#### Hidden Markov modeling to identify latent behavioral-affective states over time

A hidden Markov model (HMM)^41,42^ was used to identify latent behavioral-affective states over the year of the average student. The R *depmixS4* package (v1.4.2^43^) fit a multivariate HMM over the group-averaged time series of five variables (Stress, Negative Affect, Energy, Sleep Quality, and feeling Connected to others) to keep it manageable and interpretable. These five variables were selected a priori to represent a range of sleep, social, academic, and affective components of participants’ daily experiences. Models with one to three state solutions were fitted to test whether the HMM would reflect the three types of time-related affective and behavioral fluctuations (breaks, weekdays, and weekends) we had observed in the previous step. Model selection was performed based on lowest Bayesian Information Criterion (BIC). The identified latent states were interpreted based on the means of the five dependent variables under each state. Additionally, the year-long sequence of estimated hidden states was examined to identify meaningful structure in the presentation of the states over time.

#### Clustering analysis to identify subgroups with distinct profiles

To explore the possibility of individual differences, a latent profile analysis (LPA) assessed the presence of subgroups with distinct student profiles (see Supplementary Information for LPA technical details). LPA used the R *mclust* package (v5.4.7^44^) and included person-level means (excluding Winter Break data to focus on school-time student behavior) of the same 15 actigraphy- and daily survey-derived variables that had been selected for longitudinal examination: Sleep Duration, Sleep Regularity, Sleep Quality, Physical Activity, Energy, time spent on Schoolwork, time spent on social Interaction, Procrastination, feeling Connected to others, Negative Affect, Stress, number of Stressors, Academic Stress, Social Stress, and Status Stress. Given our relatively small sample size and to keep results interpretable, models with two to five cluster solutions were tested, and model selection was performed using BIC.

The package *mclust* offers a dimensionality reduction method similar to principal components analysis that identifies a set of linear combinations, ordered by importance as quantified by the associated eigenvalues, of the original features which capture most of the clustering structure contained in the data^45^. We used this dimensionality reduction method to generate a biplot (Fig. 5a; see more details in Supplementary Information). This approach and the means of the fitted variables for each of the identified clusters were used to describe and interpret the meaning of each cluster.

As an external validation of the LPA clustering results, we turned to independently-acquired assessments of academic performance (first-year cumulative grade point average—GPA) and psychological distress as reflected by the Global Clinical Score. The R *stats* package fitted two separate one-way ANOVAs to assess differences in person-level GPA and differences in Global Clinical Scores by the identified clusters. Following statistically significant differences, post hoc two-tailed *t*-tests were used to assess differences between cluster pairs. Statistical significance was assessed at *p* < 0.05.

#### Hidden Markov modeling across distinct student profiles

To better understand the subgroups identified by the clustering analysis, a multilevel HMM^46^ was used to examine their respective sequences of latent distress states over time, expanding the full-sample HMM approach described above. The R *mHMMbayes* package^47^ was used to fit a five-variable, three-state HMM similar to the model outlined above, but in a multilevel framework. Group-level parameters were estimated based on the cluster-averaged time series, and cluster-level parameters were subsequently sampled from the group-level distributions. Thus, while each cluster was allowed to have its own unique parameter values, these were all computed within the same HMM, with all clusters fitted by the same number and similar composition of the hidden states. The Bayesian estimation required initial values for the transition probabilities for each state and for the distribution (mean and variance) of each dependent variable in each state, and also the specification of hyperprior distributions for the dependent variables. To facilitate comparison, these values were set informed by the results from the previous, full-sample HMM. The model was run with 5000 iterations and a 500 burn-in period. The marginal probabilities of each of the three states were extracted from the model and used to compute each cluster’s most likely state on each day in the study.

#### Academic and social stressors as predictors of subsequent clinical symptoms

A final analysis performed on the first-year data used a mixed effects model to explore whether Social and Academic Stress sources predicted change in Global Clinical Scores over the year. The outcome variable was the SCL-90’s Global Severity Index (GSI) at two timepoints: end of Fall and at end of Spring, while the baseline GSI was entered as a fixed covariate. The predictors of interest were frequency of reported Social Stress sources, and frequency of reporting Academic Stress sources, entered separately per semester (Fall and Spring). Semester (Fall, Spring) and the interaction between semester and baseline symptom levels were also included as fixed effects. Random intercepts for each participant were specified in the model.

#### First-year subgroup clusters as predictors of COVID-19 clinical symptoms

To further probe the stability of the student subgroups identified in the first-year study, we assessed whether these cluster labels prospectively predicted differences in clinical symptoms under the COVID-19 follow-up study. Four separate one-way ANOVAs were used to assess differences in person-mean Global Clinical Scores, Perceived Stress, Anxiety symptoms, and Depression symptoms by the clusters identified in the first-year study, with the expectation that each of these measures should demonstrate significant effects, allowing for both prospective prediction and convergence across multiple measures. Following statistically significant differences, post hoc two-tailed *t*-tests were used to assess differences between cluster pairs. Statistical significance was assessed at *p* < 0.05 and patterns that illustrated convergence, meaning significance across multiple variables, were interpreted. These analyses were run with the R *stats* package.

## Supplementary Information

Supplementary Information.
