# Author Correction: Fluctuations in behavior and affect in college students measured using deep phenotyping

**DOI:** 10.1038/s41598-022-09563-5

**Published:** 2022-03-29

**Authors:** Constanza M. Vidal Bustamante, Garth Coombs, Habiballah Rahimi-Eichi, Patrick Mair, Jukka-Pekka Onnela, Justin T. Baker, Randy L. Buckner

**Affiliations:** 1 Department of Psychology, Harvard University, Northwest Science Building 280.05, 52 Oxford Street, Cambridge, MA 02138 USA; 2 Center for Brain Science, Harvard University, Cambridge, MA 02138 USA; 3 Department of Psychiatry, Harvard Medical School, Boston, MA 02114 USA; 4 Institute for Technology in Psychiatry, McLean Hospital, Belmont, MA 02478 USA; 5 Department of Biostatistics, Harvard T.H. Chan School of Public Health, Harvard University, Boston, MA 02115 USA; 6 Athinoula A. Martinos Center for Biomedical Imaging, Massachusetts General Hospital, Boston, Charlestown, MA 02129 USA

Correction to: *Scientific Reports* 10.1038/s41598-022-05331-7, published online 04 February 2022

The original version of this Article contained an error in the Competing interests section.

“J.P.O. is a cofounder and board member of a commercial entity, Beiwe, established in 2020, that operates in digital phenotyping. JTB has received consulting fees from Verily Life Sciences as well as consulting fees and equity from Mindstrong Health Inc. for work unrelated to the present work. All other authors declare no competing interests.”

now reads:

“J.P.O is a cofounder and board member of Phebe, a commercial entity that operates in digital phenotyping. JTB has received consulting fees from Verily Life Sciences as well as consulting fees and equity from Mindstrong Health Inc. for work unrelated to the present work. All other authors declare no competing interests.”

The original Article has been corrected.

