# Publisher Correction: Fluctuations in behavior and affect in college students measured using deep phenotyping

**DOI:** 10.1038/s41598-022-16757-4

**Published:** 2022-07-25

**Authors:** Constanza M. Vidal Bustamante, Garth Coombs, Habiballah Rahimi-Eichi, Patrick Mair, Jukka-Pekka Onnela, Justin T. Baker, Randy L. Buckner

**Affiliations:** 1 Department of Psychology, Harvard University, Northwest Science Building 280.05, 52 Oxford Street, Cambridge, MA 02138 USA; 2 Center for Brain Science, Harvard University, Cambridge, MA 02138 USA; 3 Department of Psychiatry, Harvard Medical School, Boston, MA 02114 USA; 4 Institute for Technology in Psychiatry, McLean Hospital, Belmont, MA 02478 USA; 5 Department of Biostatistics, Harvard T.H. Chan School of Public Health, Harvard University, Boston, MA 02115 USA; 6 Athinoula A. Martinos Center for Biomedical Imaging, Massachusetts General Hospital, Boston, Charlestown, MA 02129 USA

Correction to: *Scientific Reports* https://doi.org/10.1038/s41598-022-05331-7, published online 04 February 2022

The original version of this Article contained errors in Table 1, where the Metric ‘Daily survey: available observations per subject’ was incorrect for the Mean (column ‘M’) and the Median (column ‘Med’). The correct and incorrect values appear below.

Incorrect:

**Table:** 

**Metric**	**M**	**Med**
Daily survey: available observations per subject	20	21

Correct:

**Table:** 

**Metric**	**M**	**Med**
Daily survey: available observations per subject	202	216

The original Article has been corrected.

