# Supplementary material for: Fluctuations in behavior and affect in college students measured using deep phenotyping
Source: Sci Rep. 2022 Feb 4;12:1932. doi: 10.1038/s41598-022-05331-7 (PMC8816914; doi:10.1038/s41598-022-05331-7)
Supplement: Supplementary file 1 — Supplementary Information. [file 41598_2022_5331_MOESM1_ESM.pdf]

Supplemental Materials

to accompany

# Fluctuations in Behavior and Affect in College Students Measured Using Deep Phenotyping

Vidal Bustamante, Coombs, Rahimi-Eichi, Mair, Onnela, Baker, and Buckner

## Supplementary Methods: Additional Information on Smartphone-Based Daily Surveys

Each day participants received a notification on their phone screen at 5PM alerting them that a new survey was available. Participants were asked to complete all surveys consistently at a time of their choosing in the evening, after most things were done for the day but before they went to sleep. If participants did not go to sleep on a given day (e.g., pulling an all-nighter), they were asked to fill out the survey at the time they usually go to sleep. Although most items contained the wording “over the past 24 hours”, participants were told that this was not a strict 24 hours and that they should answer the items as to reflect the total time since they last answered the daily survey. However, if they had missed a survey, the “past 24 hours” wording did reflect only the past day and not all days since they last completed a survey.

## Supplementary Methods: Daily Phone-Based Survey Questions Used in Analysis

How would you gauge your energy level in the past day?
1) Little energy or motivation to do much of anything
2) Enough energy to get by but not enough to be very productive
3) Typical energy level with usual productivity
4) Plenty of energy to be even more productive than usual
5) Unusually high energy feeling hyper or even agitated at times]

How did you sleep last night?
1) Terribly: little or no sleep
2) Not so well: got some sleep but not enough
3) Sufficient: got enough sleep to function
4) Good: got a solid night sleep and felt well-rested
5) Exceptional: one of my best nights of sleep

How much were you physically active during the past 24 hours?
1) Very slightly or not at all
2) A little
3) Moderately
4) Quite a bit
5) Extremely

How much did you feel nervous or anxious over the past 24 hours?
1) Very slightly or not at all
2) A little
3) Moderately
4) Quite a bit
5) Extremely

How much did you feel excited or enthusiastic over the past 24 hours?
1) Very slightly or not at all
2) A little

3) Moderately
4) Quite a bit
5) Extremely

How much did you feel upset over the past 24 hours?
1) Very slightly or not at all
2) A little
3) Moderately
4) Quite a bit
5) Extremely

How much did you feel irritable over the past 24 hours?
1) Very slightly or not at all
2) A little
3) Moderately
4) Quite a bit
5) Extremely

How much did you feel angry or hostile over the past 24 hours?
1) Very slightly or not at all
2) A little
3) Moderately
4) Quite a bit
5) Extremely

How much did you feel happy over the past 24 hours?
1) Very slightly or not at all
2) A little
3) Moderately
4) Quite a bit
5) Extremely

How much did you feel sad or down over the past 24 hours?
1) Very slightly or not at all
2) A little
3) Moderately
4) Quite a bit
5) Extremely

How much did you feel relaxed or at ease over the past 24 hours?
1) Very slightly or not at all
2) A little
3) Moderately
4) Quite a bit
5) Extremely

How much did you feel lonely over the past 24 hours?
1) Very slightly or not at all
2) A little
3) Moderately
4) Quite a bit
5) Extremely

How much did you feel outgoing over the past 24 hours?
1) Very slightly or not at all
2) A little
3) Moderately
4) Quite a bit
5) Extremely

How much did you feel dissatisfied with yourself over the past 24 hours?
1) Very slightly or not at all
2) A little
3) Moderately
4) Quite a bit
5) Extremely

How much did you feel stressed over the past 24 hours?
1) Very slightly or not at all
2) A little
3) Moderately
4) Quite a bit
5) Extremely

Consider your sources of stress over the past 24 hours; and check all that apply:
N/A; I did not feel stressed;
Academics: homework or studying;
Academics: grades;
Academics: standing or performance compared to peers;
Academics: other;
Social relationships: family;
Social relationships: friends;
Social relationships: roommate;
Social relationships: significant other;
Social relationships: reputation;
Social relationships: social status compared to peers;
Social relationships: other;
Other: health;
Other: financial situation;
Other: extracurricular requirements;
Other: not listed

How much of your waking time did you spend interacting with others over the past 24 hours?
1) Very little of my time (0-20%)
2) Some of my time (21-40%)
3) About half of my time (41-60%)
4) Most of my time (61-80%)
5) Almost all of my time (81-100%)

How much of your waking time did you spend studying or doing homework over the past 24 hours?
1) Very little of my time (0-20%)
2) Some of my time (21-40%)
3) About half of my time (41-60%)
4) Most of my time (61-80%)
5) Almost all of my time (81-100%)

How much did you procrastinate in the last 24 hours?
1) Very little or not at all
2) A little
3) A moderate amount
4) Quite a bit
5) An extreme amount

**Supplementary Methods: Health Questionnaire**

Please specify which of the following conditions you have now or have had in the past.

| | In Your Lifetime | Currently (Now) | N/A |
|---|---|---|---|
| Cancer (other than skin cancer) | | | |
| Chronic migraine headaches | | | |
| Diabetes | | | |
| Encephalitis or meningitis | | | |
| Epilepsy | | | |
| High blood pressure | | | |
| Multiple sclerosis | | | |
| Parkinson's disease | | | |
| Stroke | | | |
| Depression (diagnosed or treated) | | | |
| Attention Deficit Hyperactivity Disorder | | | |
| Anxiety disorder | | | |
| Personality disorder | | | |
| Schizophrenia or other psychotic disorder | | | |
| Bipolar disorder | | | |
| Alcohol Abuse or Dependence | | | |
| Other Substance Abuse or Dependence | | | |
| Other significant illnesses | | | |
| Please specify | | | |

### Supplementary Methods: Additional Information on Clustering Analysis

Latent profile analysis (LPA) is a parametric clustering approach where the distribution of every observation is specified by a probability density function through a finite mixture model of G components, with each component representing a group or cluster (Fraley & Raftery, 2002; McLachlan & Peel, 2004). A popular model, and the one used in the current analysis, is the Gaussian mixture model, which assumes a (multivariate) Gaussian distribution for each component. Compared to non-parametric clustering approaches like hierarchical and k-means clustering, LPA estimates parameters subject to further statistical inference, including goodness-of-fit measures such as BIC that can be assessed to select the optimal number of clusters. Moreover, LPA performs probabilistic or "soft" cluster assignments (as opposed to deterministic or "hard" assignments like in K-means clustering), which qualifies the degree of certainty around the cluster labels. Cluster membership was assigned based on the maximum posterior probability.

We used the mclust package's dimensionality reduction method to generate a biplot in Figure 5a. The biplot (Gower & Hand, 1996) shows both the maximal separation among the clusters and the original variables used in the analysis, displayed as standardized basis vectors on a reduced 2-D subspace. The directions of the arrows represent the association of each variable with each dimension; the lengths of the vectors represent standardized regression coefficients, and the angles between them approximate the covariance structure of the variables. The variables are visualized as arrows, but they constitute axes that traverse the entire plane and onto which the participant observations (colored dots) can be projected to estimate their values on each variable.

### Supplementary Results: Additional Information on Missing Data

The first two weeks of the study had fewer observations as participants were being enrolled (**Supplementary Figure 1**). Actigraphy data showed increased missingness at the end of Winter Break reflecting the end of the devices' battery reserve, and there is a general trend for missingness to increase over the year. Across subjects, the mean Stress levels (computed as means of all days for a subject) and mean Global Clinical Scores (computed as mean of three timepoints) were not correlated with missingness rates for either actigraphy ($r = 0.13$ and $r = -0.06$, respectively; $p$s > 0.37) or survey data ($r = 0.18$ and $r = 0.06$, respectively; $p$s > 0.22). These observations suggest that participants' daily stress and global psychological distress did not introduce systematic missingness in the data. Daily rates of missing data are presented in **Figures 1** and **2**, and missing data by subject is presented in **Supplementary Figure 1.**

**Supplementary Table 1.** Results from 3-state multivariate hidden Markov model over group-averaged time series

| | **State 1 ("Lowest Distress")** | | **State 2 ("Medium Distress")** | | **State 3 ("Highest Distress")** | |
|---|---|---|---|---|---|---|
| **Dependent variable** | **Mean** | **SD** | **Mean** | **SD** | **Mean** | **SD** |
| Stressed | 1.83 | 0.24 | 2.39 | 0.18 | 2.75 | 0.20 |
| Negative affect | 1.68 | 0.07 | 1.80 | 0.08 | 1.88 | 0.10 |
| Energy | 2.80 | 0.15 | 2.88 | 0.12 | 2.87 | 0.11 |
| Sleep | 3.13 | 0.14 | 2.98 | 0.19 | 2.95 | 0.13 |
| Connected to others | 3.22 | 0.16 | 3.24 | 0.11 | 3.06 | 0.11 |

| **Transition probabilities** | **To State 1** | **To State 2** | **To State 3** |
|---|---|---|---|
| From State 1 ("Lowest Distress") | 0.91 | 0.05 | 0.04 |
| From State 2 ("Medium Distress") | 0.04 | 0.68 | 0.28 |
| From State 3 ("Highest Distress") | 0.00 | 0.18 | 0.82 |

Note: SD = Standard deviation.

**Supplementary Table 2.** Results from multilevel multivariate hidden Markov model over cluster-averaged time series

| | State 1 ("Lowest Distress") | | State 2 ("Medium Distress") | | State 3 ("Highest Distress") | |
|---|---|---|---|---|---|---|
| **Dependent variable** | **Mean** | **SD** | **Mean** | **SD** | **Mean** | **SD** |
| Stressed | 1.50 | 0.27 | 2.30 | 0.32 | 3.00 | 0.28 |
| Negative affect | 1.50 | 0.17 | 1.80 | 0.16 | 2.20 | 0.19 |
| Energy | 2.51 | 0.22 | 2.81 | 0.20 | 2.99 | 0.22 |
| Sleep | 3.20 | 0.30 | 3.00 | 0.23 | 2.80 | 0.24 |
| Connected to others | 3.50 | 0.27 | 3.29 | 0.24 | 3.00 | 0.20 |
| **Transition probabilities** | **To State 1** | **To State 2** | **To State 3** | | | |
| From State 1 ("Lowest Distress") | 0.70 | 0.22 | 0.07 | | | |
| From State 2 ("Medium Distress") | 0.11 | 0.83 | 0.05 | | | |
| From State 3 ("Highest Distress") | 0.13 | 0.23 | 0.61 | | | |

Note: SD = Standard deviation.

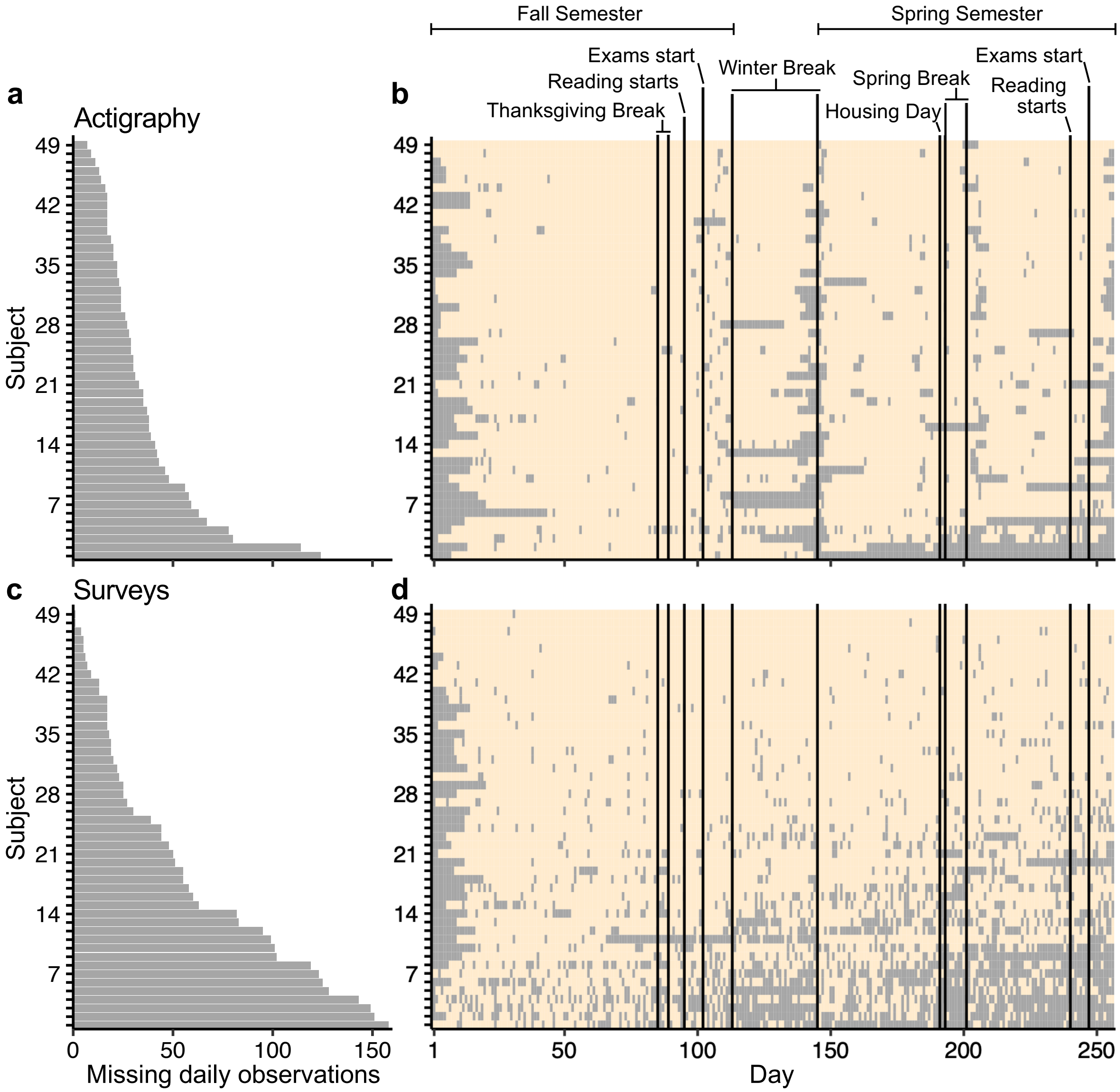


**Supplementary Figure 1. Relative to actigraphy (passive) data, missingness in daily survey (active) data was greater and increased over the course of the year.** Missing daily observations per subject in actigraphy (**a, b**) and survey data (**c, d**). The y-axis ranks subjects bottom-top from having most to least missing data in that category (actigraphy or survey). Panels **b** and **d** show daily observations over the study period colored by whether they were available (cream color) or missing (grey color). Landmark events in the academic calendar are indicated by vertical lines, labeled at the top of **b**.

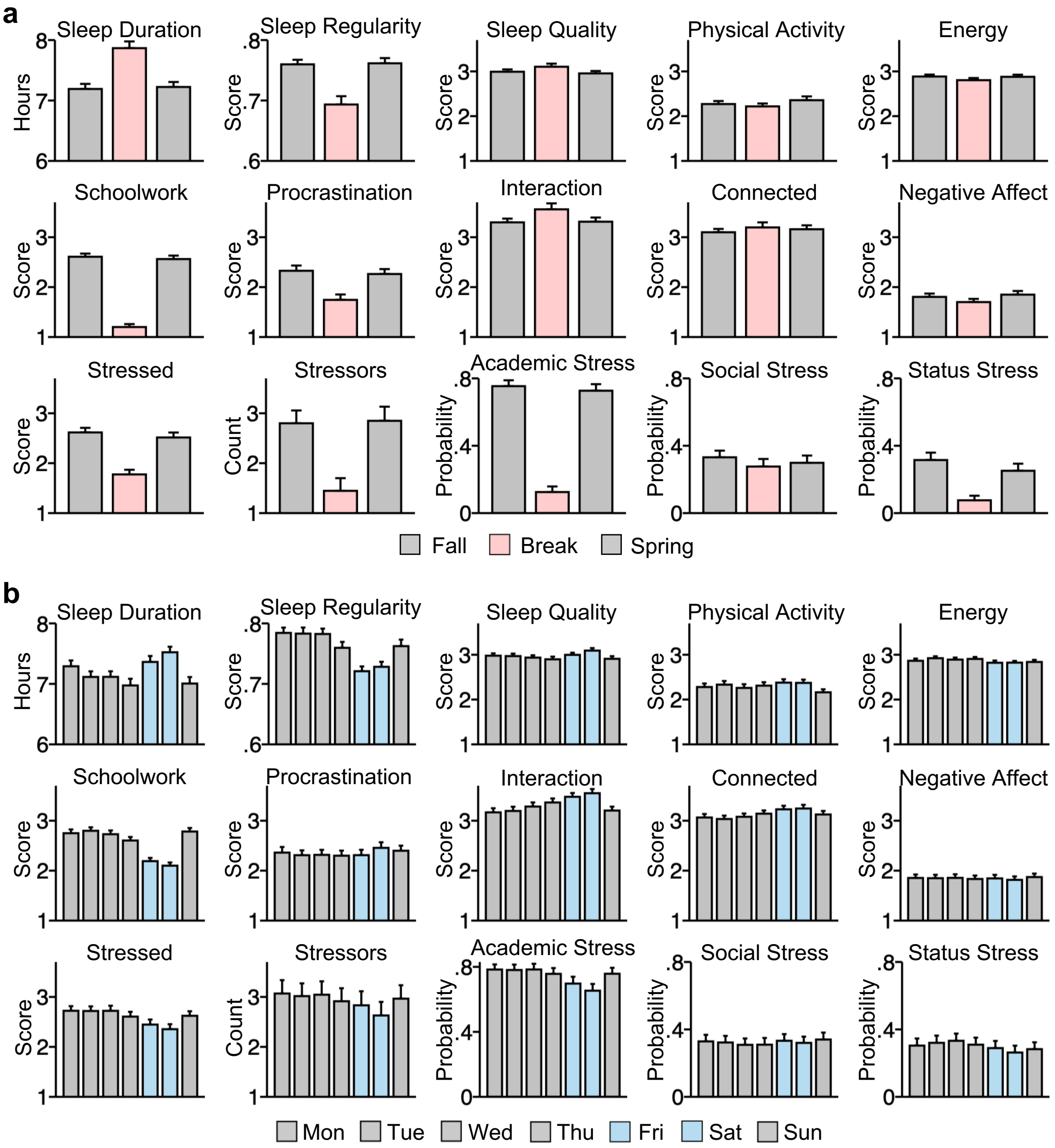


**Supplementary Figure 2. a. During Winter Break, the average student had longer Sleep Duration, spent less time on Schoolwork and more on social Interaction, and felt less Stressed relative to the Fall and Spring school semesters.** Bar plots show between-subject means separately by school semesters (Fall and Spring) and Winter Break. Error bars indicate the standard error of the mean. **b. The differences in behavior and affect between school semesters and Winter Break are recapitulated by weekdays and weekends within the school semesters.** Bar plots show between-subject means separately by day of the week. Sleep-related metrics correspond to night when participant went to sleep. Winter Break observations were not included. Error bars indicate the standard error of the mean.

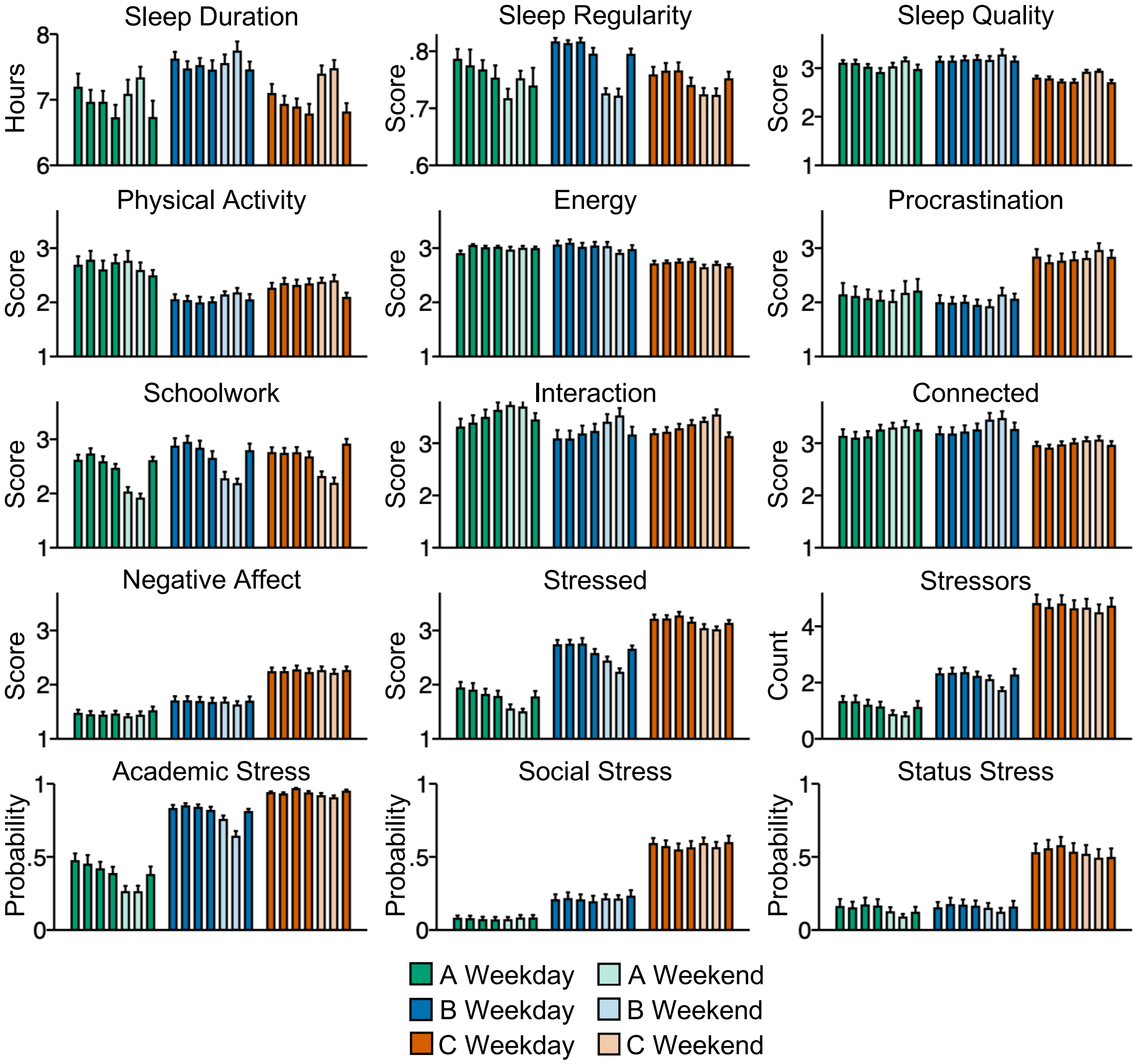


**Supplementary Figure 3. Week-related fluctuations in behavioral and affective metrics are conserved within all three student profiles, but Cluster C fared overall worse than other groups even during weekends.** Bar plots show between-person means of each variable separately by student profile and by day of the week. Error bars represent standard errors of the mean.

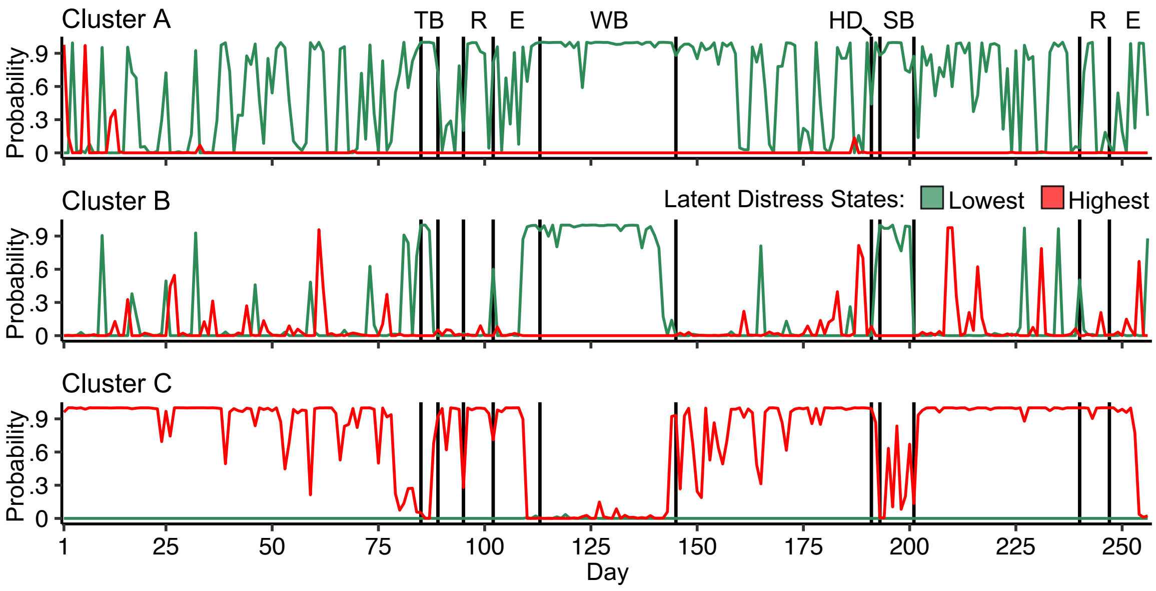


**Supplementary Figure 4**. **Student cluster features are recapitulated by their Latent Distress State probabilities across the year.** Marginal probabilities of Lowest and Highest Latent Distress States for each of the three student Clusters. TB = Thanksgiving Break; R = Reading period; E = Exams period; WB = Winter Break; HD = Housing Day; SB = Spring Break.

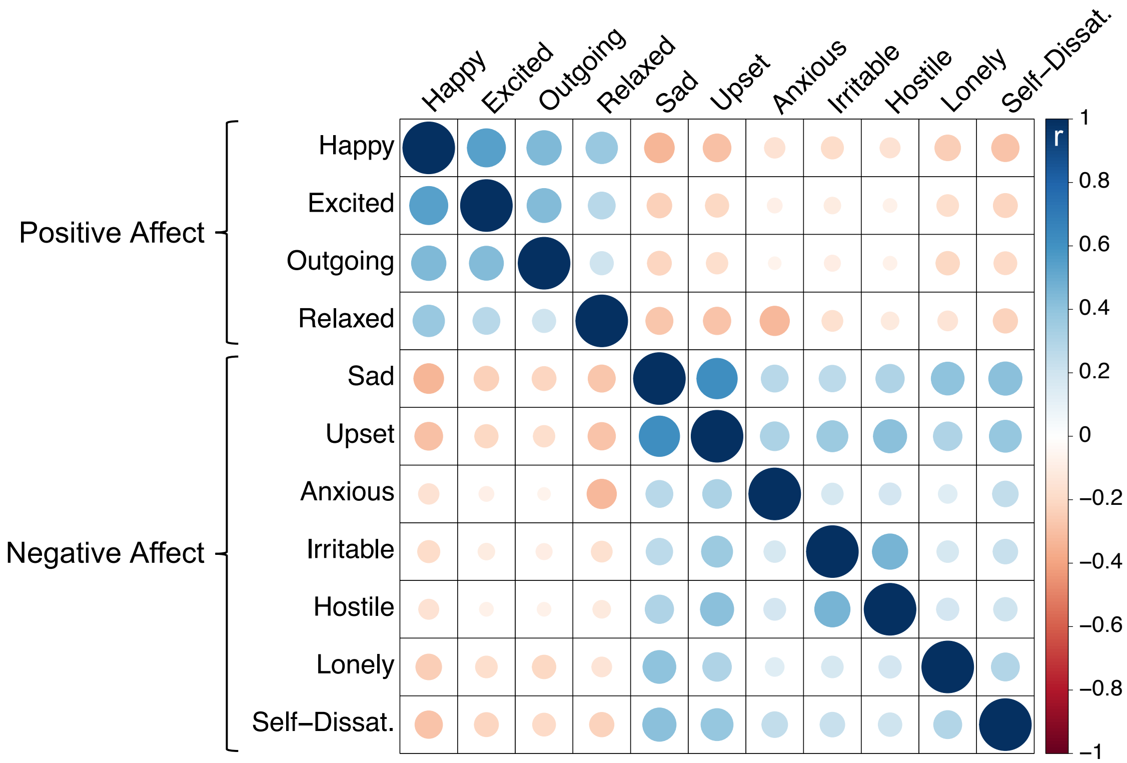


**Supplementary Figure 5. Individual items within affect composite scores had positive same-day correlations.** Within-subjects Pearson correlation matrix for affect items included in the daily survey. Labels on the left indicate the individual items that went into Positive Affect and Negative Affect composite scores. r = Pearson correlation coefficient.
